# Adjuvant Autologous Melanoma Vaccine for Macroscopic Stage III Disease: Survival, Biomarkers, and Improved Response to CTLA-4 Blockade

**DOI:** 10.1155/2016/8121985

**Published:** 2016-05-18

**Authors:** Michal Lotem, Sharon Merims, Stephen Frank, Tamar Hamburger, Aviram Nissan, Luna Kadouri, Jonathan Cohen, Ravid Straussman, Galit Eisenberg, Shoshana Frankenburg, Einat Carmon, Bilal Alaiyan, Shlomo Shneibaum, Zeynep Ozge Ayyildiz, Murat Isbilen, Kerem Mert Senses, Ilan Ron, Hanna Steinberg, Yoav Smith, Eitan Shiloni, Ali Osmay Gure, Tamar Peretz

**Affiliations:** ^1^Sharett Institute of Oncology, Hadassah Hebrew University Hospital, Ein Karem Campus, 91120 Jerusalem, Israel; ^2^Departments of Surgery, Hadassah Hebrew University Hospital, Mount Scopus Campus, 91240 Jerusalem, Israel; ^3^Department of Molecular Cell Biology, Weizmann Institute of Science, 7610001 Rehovot, Israel; ^4^Department of Surgery, Tel Aviv Sourasky Medical Center, 64239 Tel Aviv, Israel; ^5^Department of Molecular Biology and Genetics, Bilkent University, 06800 Ankara, Turkey; ^6^Department of Oncology, Tel Aviv Sourasky Medical Center, 64239 Tel Aviv, Israel; ^7^Genomic Data Analysis Unit, Hebrew University Medical School, 91120 Jerusalem, Israel; ^8^Department of Surgery, Bnai Zion Medical Center, 31048 Haifa, Israel

## Abstract

*Background*. There is not yet an agreed adjuvant treatment for melanoma patients with American Joint Committee on Cancer stages III B and C. We report administration of an autologous melanoma vaccine to prevent disease recurrence.* Patients and Methods*. 126 patients received eight doses of irradiated autologous melanoma cells conjugated to dinitrophenyl and mixed with BCG. Delayed type hypersensitivity (DTH) response to unmodified melanoma cells was determined on the vaccine days 5 and 8. Gene expression analysis was performed on 35 tumors from patients with good or poor survival.* Results*. Median overall survival was 88 months with a 5-year survival of 54%. Patients attaining a strong DTH response had a significantly better (*p* = 0.0001) 5-year overall survival of 75% compared with 44% in patients without a strong response. Gene expression array linked a 50-gene signature to prognosis, including a cluster of four cancer testis antigens: CTAG2 (NY-ESO-2), MAGEA1, SSX1, and SSX4. Thirty-five patients, who received an autologous vaccine, followed by ipilimumab for progressive disease, had a significantly improved 3-year survival of 46% compared with 19% in nonvaccinated patients treated with ipilimumab alone (*p* = 0.007).* Conclusion*. Improved survival in patients attaining a strong DTH and increased response rate with subsequent ipilimumab suggests that the autologous vaccine confers protective immunity.

## 1. Introduction

The treatment of metastatic melanoma has been revolutionized in the last three years, with the FDA registration of Yervoy*™*, a monoclonal antibody blocking lymphocyte regulatory receptor cytotoxic T lymphocyte-associated antigen 4 (CTLA-4), and shortly afterward, the entry to the clinic of Zelboraf, a small molecule inhibitor of mutated B-RAF. From being an incurable disease, stage IV melanoma has become an illness in which prolonged and even complete responses can be envisioned. However, while the prospects have improved for stage IV disease, for patients with American Joint Committee on Cancer (AJCC) stage III disease no new treatment options have been developed and validated since the approval of interferon *α* (IFN*α*) almost two decades ago [1–7]. A pegylated formulation did not offer improved tolerability and treatment was discontinued due to toxicity. Both the EORTC trials and the ECOG pooled analysis [8] showed that AJCC stage III patients with macroscopic lymph node involvement derived the smallest survival benefit, if any, from IFN*α*.

In this situation, melanoma vaccines could have been an alternative to IFN*α*, since they could induce a tumor-specific immune response to inhibit micrometastases at a stage when the suppressive effects of an advanced tumor are not yet an obstacle [9]. In the few controlled clinical trials reported to date, vaccinated patients did not experience a survival benefit [10]. This fact, together with the paucity of objective responses to active immunization in stage IV melanoma patients using different vaccination strategies, rendered a general impression of the futility of cancer vaccines [11, 12].

The resurgence of interest in cancer vaccination was the result of several clinical trials demonstrating that a component of active immunization could improve clinical outcome of immunotherapy protocols. Examples include the addition of a peptide vaccine to the administration of high dose interleukin-2 (IL-2) [13] and the use of a GMCSF-secreting tumor vaccine in combination with CTLA-4 blockade for metastatic prostate cancer [14]. These trials led to the increasing understanding that cancer immunotherapy is a multifaceted strategy and that a single treatment modality would not suffice. Noting that individuals exhibit heterogeneity of tumor antigens [15], the use of autologous tumor as a basis for vaccination can provide antigen authenticity. The unique expression profile of normal and mutated proteins in the patient's tumor cells is presented in conjunction with their own major histocompatibility complex (MHC) molecules, and this combination is necessary to induce antigen-specific reactive lymphocytes [16]. The coadministration of Bacillus Calmette-Guérin (BCG), a widely used immunological adjuvant, with the autologous vaccine, has previously been shown to enhance response to autologous vaccination protocols [17, 18].

In this paper we report our experience with an autologous melanoma vaccine as an adjuvant therapy for melanoma patients in the advanced categories of AJCC stage III disease: macroscopic lymph node involvement and resectable in-transit metastases. We demonstrate that clinical immune response (delayed type hypersensitivity, DTH) is linked to improved survival. Furthermore, using this vaccination protocol, molecular analysis of the melanoma showed that cancer testis antigens (CTAs), which are generally considered targets of immune response, are predictive of survival. Interestingly, patients who had previously received the melanoma cell vaccine had improved overall survival when treated with ipilimumab immunotherapy for metastatic disease.

## 2. Materials and Methods

### 2.1. Patients

This prospective phase II, single-institution, single-arm study included patients with operable AJCC stages IIIB and C melanoma. Clinical staging was based on palpable lymph nodes and/or satellites prior to surgery. Since we did not have pathological data on ulceration of the primary melanoma for all patients, AJCC stage IIIB was defined for any T (tumor) with pathological N1b, N2b, and N2c. Stage IIIC was defined as any T with pathological N3 [19].

### 2.2. Outcomes

The aim of the study was to document overall and disease-free survival (primary outcome) and to correlate skin reactivity to the autologous tumor of patients that were treated with the autologous melanoma cell vaccine with survival (secondary outcome).

### 2.3. Inclusion and Exclusion Criteria

To participate, patients had to undergo complete removal of their metastatic disease and have a normal CT scan within 30 days prior to vaccination. Additional inclusion criteria included an age of 18 years or older, normal liver and renal function tests, baseline lactate dehydrogenase (LDH) value below the laboratory upper limit of normal, and ability to provide informed consent. Exclusion criteria included primary ocular melanoma. Fertile patients were requested to use adequate anticonceptive measures throughout the study period.

Screening procedures included baseline blood analyses (hematological, chemical) and computed tomography (CT) and/or magnetic resonance imaging (MRI) of the entire body, including the brain, every 4 months in the first 2 years and then every 6 months for 10 years. The study was not sponsored and was conducted following approval by the institutional ethics committee; written informed consent was obtained from all patients. Seventy-five patients from this group were included in an immune monitoring study reported earlier [16].

### 2.4. Melanoma Cell Lines

For the preparation of the autologous vaccine, melanoma cell lines were established from resected metastases. All patients gave their informed consent to receive the vaccine. The melanoma cell lines were established and cultured as described [18]. Briefly, cells were extracted mechanically from fresh and sterile tumor specimens, frozen, and stored in liquid nitrogen in a medium containing 2.5% human albumin and 20% DMSO until needed. To assure melanocytic progeny, the expressions of S100, MART-1, and gp100 were determined by immunostaining using polyclonal rabbit anti-S100, monoclonal A-103, and HMB-45 Abs, respectively (Dako). Positive staining of more than 50% of cells with at least one of these antibodies was required. MHC class I-related chain A (MICA) expression was determined by flow cytometry of melanoma cell lines using anti-human MICA-APC (Allophycocyanin), R&D systems, Minneapolis, MN, USA.

Cell lines were routinely tested for mycoplasma contamination by EZ-PCR (Biological Laboratories, Beth Haemek, Israel), according to the manufacturer's instructions. Tumor cultures that were found contaminated were incubated in the presence of 10 mg/mL Ciproxin 200 (Bayer) for two weeks, with change of medium every three days. The cells were retested after treatment and were used only after being found mycoplasma-free.

### 2.5. Vaccine Preparation and Vaccination Procedure

Melanoma cell lines were expanded to the required number necessary for preparation of at least 8 vaccine doses of 10–25 × 10^6^ cells each and cryopreserved at −70°C. On the day of treatment, one dose of cells was thawed, washed, and irradiated to a dose of 230 Gy. At this stage, cells were still viable. Conjugation of melanoma cells with DNP was performed as described [20], leading to death of the cells (as determined by trypan blue exclusion). BCG (Statens Serum Institut, Denmark) was added to the vaccine prior to injection, diluted to 1 : 50 for the first three vaccine doses and up to 1 : 500 for the following doses, to avoid overreactivity at the injection site. BCG was reported to trigger dendritic cell maturation and to aid in diverting the CD4 T cell response towards a Th1 phenotype [21]. The vaccination procedure was carried out as described [18]. Briefly, patients were sensitized to DNP, to enhance the response to the vaccine, by applying 0.1 mL of 2% DNP dissolved in acetone-corn oil (Sigma) topically to the inner aspect of the arm ten days prior to injection of the first vaccine. Cyclophosphamide, 300 mg/m^2^ per dose, was given 4 days preceding the first and second vaccines. This practice was based on the observation that cyclophosphamide prior to vaccination can reduce the proportion of T regulatory cells and enhance tumor-specific immune response [22–25]. Generally, vaccines were injected in 3 adjacent sites on the upper arm or thigh, avoiding limbs with dissected lymphatic basins. Each patient received eight vaccine doses, at three-week intervals.

Patients were evaluated periodically every 3 months and had a total body CT scan performed every 6 months, or as required according to their symptoms.

### 2.6. DTH Reaction

Evaluation of DTH to autologous melanoma cells was performed on the vaccine days 5 and 8, by intradermal injection of 1–3 × 10^6^ irradiated (170 Gy) autologous melanoma cells. The DTH response was measured 48 h after injection. Erythema diameter of 5 mm and less was arbitrarily defined as negative; 5–10 mm weak; 10–15 mm positive; and ≥15 mm strong positive DTH.

### 2.7. Gene Expression and Connectivity Map Analysis (C-MAP)

Thirty-five melanoma cell lines that were retrieved from 35 vaccinated patients were selected for gene expression analysis based on retrospective survival data of the donors. Gene expression profiling was performed using an assay based on a collection of cellular genomic signatures that produced a pattern-matching tool and formulation-based deduction of a wider expression profile. One thousand transcripts were identified, from which the remainder of the transcriptome could be computationally inferred. These 1,000 “landmark” transcripts were measured on Luminex beads, as part of the Connectivity Map (C-MAP) project (unpublished, R. Narayan, Broad Institute of Harvard University and MIT, Cambridge, MA). Cultures underwent a median of 4 passages (range 2–13) and mRNA was extracted from melanoma cell microcultures harvested at 90% to 100% confluence, produced in a synchronous way, under identical conditions of growth, in four replicates. Initial analysis was conducted with a bioconductor- (R-) based test (SSAT), which applies a Cox-regression analysis followed by a second test based on rank statistics. The analysis determines the best cut-off value that separates patients into those with favorable versus worse survival time.

### 2.8. Quantitative Real Time PCR (qRT-PCR)

Applied Biosystems premade and custom primer probes designed with NCBI Primer–Blast Tool were used (http://www.ncbi.nlm.nih.gov/tools/primer-blast/). RNA was normalized to GAPDH-RNA content using ABI 7500 SDS software, v1.2.2 (Applied Biosystems Inc., Foster City, CA). Positive and negative controls, as well as samples with no DNA, were included in every qRT-PCR experiment. PCR reactions were performed using ABI qRT-PCR thermocycler (7500 Real Time PCR System, Applied Biosystems Inc., Foster City, CA). The qRT-PCR program was run for 45 cycles, following an initial incubation at 95°C, 10 min. Each cycle consisted of 95°C × 15 sec and 60°C × 1 min.

*B-RAF genotype* was determined using Cobas® 4800 (Roche).

### 2.9. Patients Undergoing Ipilimumab Treatment

During the autologous vaccine study period, ipilimumab (Yervoy, BMS) was administered in our institution to patients with metastatic melanoma in the framework of BMS studies CA184-004, 024, and 025. Since its FDA approval as a standard second-line treatment, ipilimumab was given after a one- or two-dose course of DTIC. Patients from the autologous vaccine study which later developed nonresectable metastatic disease were among a larger group recruited for these protocols. Survival data is reported for all patients getting ipilimumab during 2007–2014.

### 2.10. Statistical Method

The comparison of survival curves between groups was carried out using the Kaplan-Meier Survival analysis with the log rank test. All tests applied were two-tailed, and a *p* value of 5% or less was considered statistically significant.

## 3. Results

### 3.1. Study Patients

Melanoma metastases were obtained from 159 eligible patients. From 33 patients (20%) we could not generate the number of cells required for the treatment. A total of 126 patients (55% male; median age, 59 years) with postoperative AJCC stages IIIB and C (45% stage IIIB; 55% stage IIIC) were enrolled. For patient characteristics see Table 1. Twenty-four patients (19%) presented with enlarged lymph nodes (LNs) at the time of diagnosis of the primary melanoma; 11 (9%) had unknown primary lesion; 22 (17%) had metastases* in transit*. Nineteen patients (15%) had noninvolved sentinel LN but developed macroscopic disease later, in the same lymphatic basin. Forty-two patients (33%) had not undergone a sentinel LN biopsy and developed macroscopic LN involvement. The mean number of involved lymph nodes was 2.4, ranging from 0 (satellites) to 17. Fifty-six patients (44%) had undergone radiotherapy in addition to the surgical procedure, which was added when more than three nodes were involved or in cases of extracapsular invasion by melanoma cells.

### 3.2. Vaccine Safety

No grade 3-4 adverse events (CTCAE V4) were encountered among the 126 vaccinated patients. In all patients, an erythematous nodule developed at the vaccination site and resolved in the course of 3–6 months leaving a depressed scar.

### 3.3. Patient Survival Correlates with Intensity of Evolving DTH Response to Unmodified Melanoma Cells

**Figure 1:**
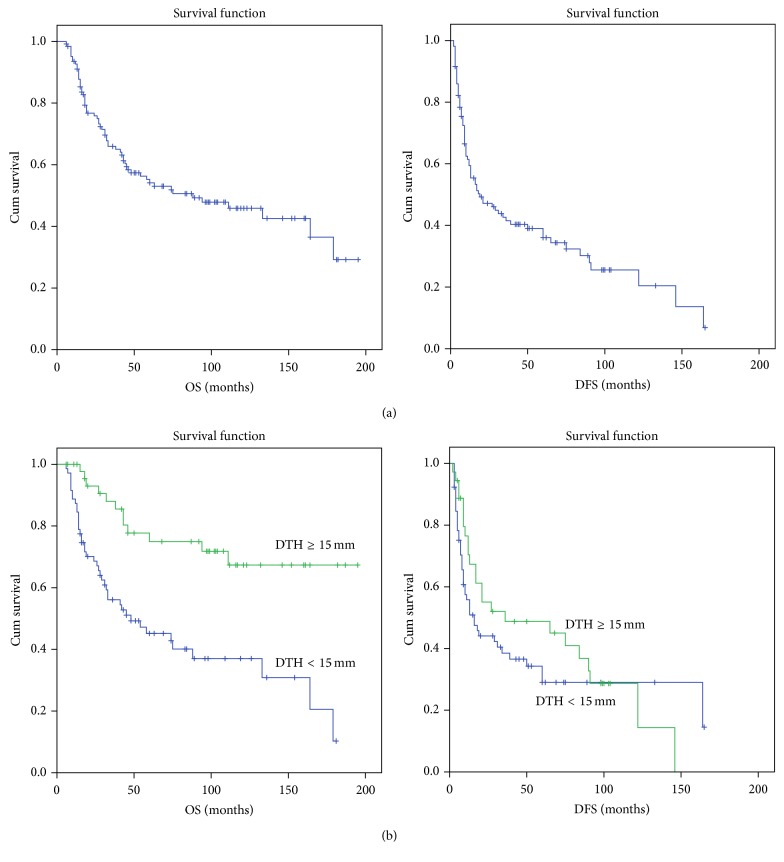
Kaplan-Meier survival curves of 126 melanoma patients with AJCC stages III B and C disease. (a) Survival data of all patients undergoing autologous vaccination. (b) Correlation of survival with delayed type hypersensitivity (DTH) response to unmodified melanoma attained following vaccination. OS: overall survival; DFS: disease free survival.

The OS and disease-free survival (DFS) of participating patients were measured from the day of the first vaccine until the current analysis was performed. A total of 126 patients were included, with a median follow-up of 44.5 months (range 8–189 months). OS survival data was available for all 126 patients and DFS data was available for 107 patients. OS and DFS were analyzed for DTH < 15 mm (weak/positive DTH) versus DTH > 15 mm (strong positive DTH). Overall, for the whole cohort, the 5-year OS was 54% and DFS was 34%. There was no difference between OS of stage IIIB and IIIC patients (*p* = 0.182). Of 119 patients with recorded DTH response, 48 patients (40%) attained strong positive DTH (DTH > 15 mm), whereas 71 (60%) had a weak DTH response (<15 mm). The patients with strong DTH response had a 5-year OS of 75% and DFS of 47%. In contrast, patients with weak DTH had a significantly lower 5-year OS of 44% (*p* < 0.0001) and DFS of 26% (*p* = 0.27). Using the Kaplan-Meier analysis and the log rank test, the single parameter that most strongly correlated with OS and DFS in a univariate analysis was the DTH response (Figure 1). In Table 3, OS and DFS are compared between patients attaining DTH responses of 10 and 15 mm and patients who did not develop such response. For patients who attained strong positive DTH (>15 mm), the 5-year overall survival hazard ratio (HR) was 0.24 (95% CI 0.1–0.53; *p* < 0.001). The HR for 5-year disease recurrence was 0.4 (95% CI 0.1–0.83, *p* = 0.015 Pearson's chi square test), but in a longer follow-up, the protection from recurrence decreased to a HR of 0.63 (95% CI 0.3–1.32; *p* = 0.2). Age, gender, and depth of invasion of the primary melanoma had no impact on survival. In a survival analysis done for DTH cut-off of 10 mm, a similar trend was noted with a smaller *p* value (0.003) for improved 5-year OS in patients attaining a DTH response of >10 mm (64%) versus 32% in patients with DTH <10 mm. DFS was similar in the two groups (*p* = 0.36). Thus, the acquisition of powerful skin reactivity against nonmodified autologous melanoma cells, which reflects the development of specific cell mediated immunity, correlates favorably with survival, supporting previous results by us and by others, for example, [16, 18, 26].

For 56 patients in whom more than 3 involved lymph nodes were removed, radiation therapy was added to the resected lymphatic basin. Even though the patients requiring this added treatment belonged to a grave prognostic group, radiotherapy may enhance the immune response of the patients [27]. Indeed, the rate of strong DTH response in patients receiving radiotherapy was 42%. Five-year OS of these patients was 58% compared to 33% of those who received radiotherapy and had a weak DTH response (*p* = 0.024).

### 3.4. Cancer Testis Antigen mRNA Expression in Melanoma Cells Correlates with Improved OS

The C-MAP project was based on a collection of cellular genomic signatures to drugs, disease states, and cancer, in order to produce a pattern-matching tool and a formulation-based deduction of a wider expression profile. One thousand transcripts were identified from which the remainder of the transcriptome could be computationally inferred. These 1,000 “landmark” transcripts were measured on Luminex beads (unpublished, R. Narayan, Broad Institute of Harvard University and MIT, Cambridge, MA).

**Figure 2:**
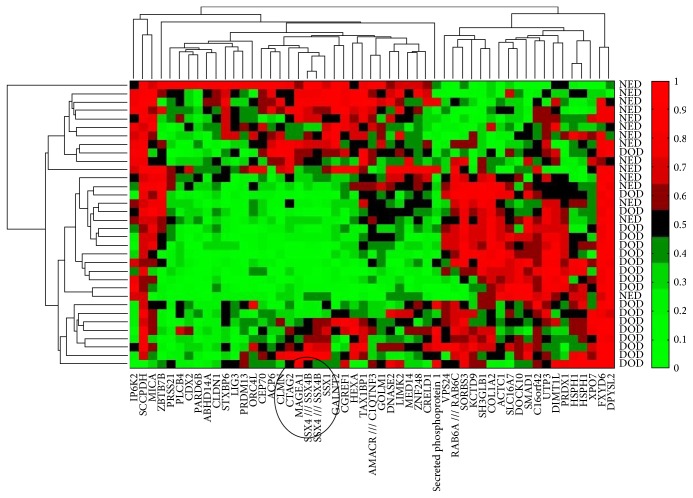
Hierarchical clustering gene expression of a 50-gene signature showing strongest association with prognosis in 35 stage III melanoma patients. Cancer testis antigens CTAG2, MAGEA1, SSX1, and SSX4 are circled in a cluster. NED: no evidence of disease at time of analysis; DOD: died of metastatic disease.

**Figure 3:**
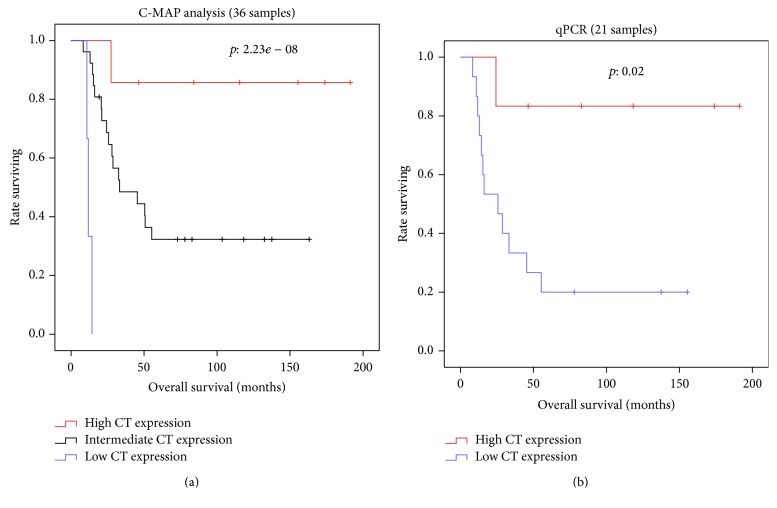
Overall survival curves stratifying the patients according to integrated cancer testis antigen genes expression. As CTA genes are coexpressed, we performed a principal component analysis (PCA) of the genes to stratify the patients into low/intermediate and high expression based on first principal component (PC1) values. (a) PC1 was determined in 35 melanoma lines based on all 51 probe sets from CTA genes in the C-MAP array. (b) PC1 was determined in 21 melanoma lines based on qPCR data generated for CTAs MAGE-A1, SSX1, SSX4, and NY-ESO-1 (CTAG1B).

Thirty-five tumor samples, representing distinct subclasses of poor and good responders, were selected for C-MAP analysis: (1) eighteen poor responding patients with a median OS of 19 months (range 8–34), all of whom failed to develop strong skin reactivity to their autologous tumor, and (2) seventeen good responding patients with median OS of 105 months (range 46–194), 12 of whom also developed strong skin reactivity (DTH data missing for one).  Figure 2 shows the hierarchical clustering of 50 genes expressed on patients' melanoma cells, which yield a significantly improved or worsened HR for survival. Several genes of interest are listed in Table 4. Cancer testis antigens CTAG2 (NY-ESO-2), MAGEA1, SSX1, and SSX4 clustered together in the hierarchical diagram (depicted in a circle in Figure 2). High expression of each of these CTA genes was associated with a reduced risk of death. As CTA genes are coexpressed, we performed a principal component analysis (PCA) of the genes to stratify the patients, using the “princomp” function in R. We found the first principal component (PC1) to explain more than 80% of the variance when expression values from the 35 samples corresponding to all 51 probe sets from CTA genes in the C-MAP array were used (Figure 3(a)). To validate the predictive data extracted from C-MAP, we used qPCR results obtained from 21 melanoma line samples for MAGE-A1, SSX1, SSX4, and NY-ESO-1 (CTAG1B). Similar to the in silico data, a PCA analysis based on these four genes was able to explain 75% of the variance. In both analyses, stratifying the patients into low/intermediate and high expression based on PC1 values, we show worse outcome for low CTA expressors (below quartile 1) compared to high expressors (above quartile 4), *p* = 0.02 (Figure 3). A similar analysis performed using an unrelated melanoma cohort (GSE19234 [28]), which never had an autologous vaccine, did not reveal an association between CTA gene expression and survival (not shown), suggesting that CTA gene expression is not a prognostic factor by itself but becomes one in the context of autologous vaccination.

**Figure 4:**
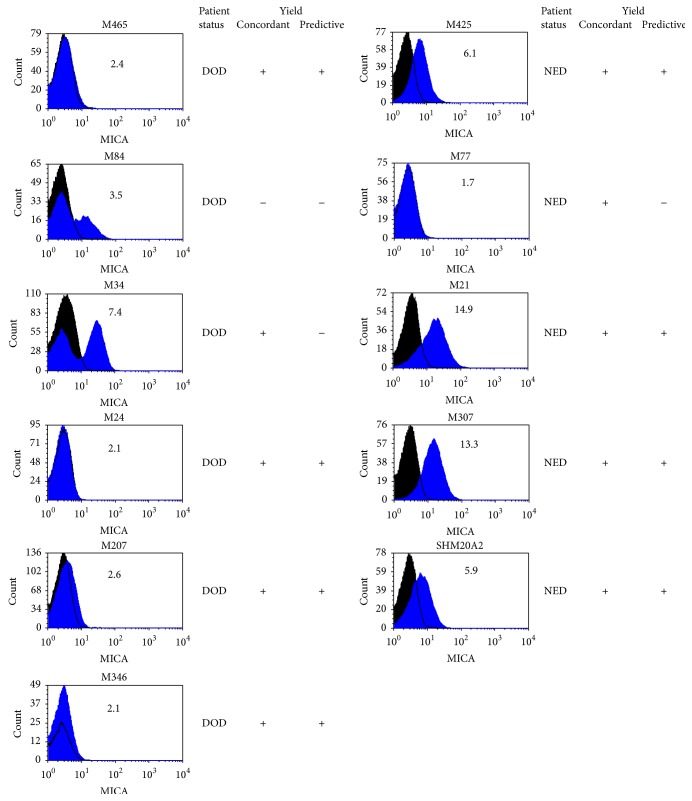
Flow cytometry analysis of MICA surface expression on melanoma cell lines. The number in the histogram is the Luminex 1000 expression value of MICA. Black histogram: background staining with isotype control Ab. Blue histogram: MICA staining with anti-MICA specific Ab.

MICA (MHC class I related chain A), another gene which correlated with a reduced risk of death and for which there is an antibody for flow cytometry, was used to validate the expression data (Figure 4). Out of 11 samples analyzed, 10 yielded MICA protein expression (by flow cytometry) concordant with the gene expression value. In 8 samples the data was predictive of patient's current status, whether alive, no evidence of disease (NED), or died of disease (DOD).

### 3.5. B-RAF Status and Survival

B-RAF status was determined for 32 patients of the 35 that were analyzed for gene expression. Eleven patients (33%) harbored the V600E mutation. The median survival for patients with V600E mutation was 50 months compared with 45 months for the wild type (WT) group (*p* = 0.9). Since most patients in the series who had recurrent disease died before 2012, none of them were treated with B-RAF inhibitors when they developed metastases, and consequently differences in survival could not be attributed to better treatment options. Four out of 11 patients with V600E mutations had strong DTH response (36%), compared with seven out of 21 (33.3%) in the WT group (*p* = 0.86).

### 3.6. Patients Who Received Melanoma Vaccine Had Improved Survival following Ipilimumab Treatment for Advanced Disease

Thirty-five patients who received melanoma cell vaccine and developed nonresectable metastatic disease were referred to ipilimumab treatment in BMS studies CA 184-004, 024, and 025 and later as a standard second-line treatment. These patients were compared with a nonselected concurrent group of other 35 patients, who never received the vaccine (Table 2). The majority, 62 patients (89%), received ipilimumab at a dose of 3 mg/kg. Response Evaluation Criteria in Solid Tumors (RECIST) were used to define response to treatment. Complete response was achieved in six patients (8.6%), partial response in 14 (20%), and stable disease in 7 (10%). Median OS of the group was 14 months (95% CI 5.7–22 months). Multivariate analysis revealed that the single parameter that correlated with an improved OS time was previous treatment with melanoma cell vaccine. In the group of 35 vaccinated patients, median OS was 31 months with a 3-year survival of 46%, compared with a median OS of 9 months and 3-year survival of only 19% in the nonvaccinated patients, (*p* = 0.007, Pearson's chi square test). The results from this retrospective cohort of patients suggest that response rate and survival are improved when ipilimumab treatment succeeds autologous melanoma vaccine.

## 4. Discussion

In this phase II study we administered a vaccine composed of the autologous tumor given to melanoma patients as a postoperative adjuvant for AJCC stage III disease following resection of macro metastases. The study was single-armed, as it was initiated prior to the registration of IFN*α* as standard of care for the adjuvant treatment of melanoma stages IIB and III. In an initial cohort, which included patients of worse prognoses (stages IIIB, IIIC, and IV) we observed an unexpected good survival rate with this vaccine [18]. In view of the reduced toxicity of the vaccination regimen, we did not offer patients a high dose interferon arm after its registration. On the other hand, the inclusion of an observation arm was ethically questionable. Thus, we opted to continue with the protocol in its single-arm design, to record survival rates in adjuvant stage IIIB and C patients. Eventually, these patients were the less likely ones to derive benefit from adjuvant IFN*α* therapy, since treatment with the standard of care IFN*α*-2b or PEG-IFN did not yield any survival advantage for them [29]. The projected 5-year OS for these patients was estimated at 40% and the DFS at 30%, as shown in the Kaplan-Meier curves generated in a meta-analysis from EORTC trials 18952 and 18992 of patients with AJCC stage III-N2. In our group of patients with the same stage disease, the 5-year OS reached 54%, with a median OS of 88 months and a 5-year DFS of 34%; these were achieved with no major adverse effects. In another series, the Nordic study, the best median survival, 72 months, was derived from intermediate-high doses of IFN*α*-2b for one year. These results, worse than those we observed with the autologous vaccine, were generated in a group of patients in which 35% had earlier stage disease (IIIA).

Notably, in our study population, patients with a strong positive skin reaction to their unmodified melanoma had much better prognosis, with a 5-year OS of 75% and DFS of 44%. The link between immune parameters that attest to the development of antitumor response and improved survival has been previously observed [17, 30–36]. DTH is a crude measurement, but an easy test to apply on all patients. Several vaccine clinical trials included a DTH test as part of their clinical evaluation and reported a positive correlation between DTH response and longer survival [37, 38]. Biopsies taken from skin injection sites revealed vaccine-induced antigen-specific T cells [39, 40]. We previously demonstrated effective antimelanoma CD4 T cell activity associated with improved survival in a cohort included in the present study [16]. Thus, a positive DTH reaction could be indicative of the emergence of antimelanoma immunity, and the improved survival of patients attaining strong DTH would attest to the vaccine's protective effects.

Since autologous tumor tissue is often not available for vaccine preparation, defined antigens, consisting of short or long peptides, are used as a substitution [41–43]. The enthusiasm for the use of defined antigens decreased when successful generation of antigen-specific T cells failed to protect against tumor progression [11, 44]. Loss of MHC and impaired peptide presentation by the melanoma were among the reasons for vaccine failure, but another limitation is that many tumor-progeny antigens are essentially self-antigens that evoke weak responses [45]. Unlike wildtype proteins, mutations have the potential to generate neoantigens which are better targets. The significance of mutation-derived neoantigens was illustrated when adoptively transferred tumor-infiltrating lymphocytes that destroyed melanoma in patients were surveyed and found to target mutated epitopes [46]. Furthermore, recent data has clearly demonstrated that these mutations are, in large part, the functional targets of immune checkpoint blockade [47]. It may not take long until “mutanome” libraries are generated for melanoma patients. But until the hurdles of preparing a totally individualized vaccine are overcome, the best source of mutated antigens, as we have previously shown, is still the autologous tumor and, ideally, those tumors that express both MHC class I and class II [16].

Another important component of melanoma cell vaccines, as reflected by our data, are a class of antigens known as cancer testis antigens. CTAs are products of several multigene families, many of which map to chromosome X, that have arisen through chromosomal duplications and were initially identified through immunologic assays [48]. When associated with disease outcome, CTAs sometimes confer worse prognosis [49], but when protective immune responses are recorded, CTAs are dominant targets. For example, rising antibody responses to CTA NY-ESO-1 (CTAG1B) were recorded following shrinkage of melanoma in a patient with abscopal tumor response [50], and among patients treated with ipilimumab, seropositivity to NY-ESO-1 with associated CD8 T cell response correlated with 77% clinical benefit [51].

Using a Luminex-based method to generate a gene expression array and qPCR validation, we showed increased expression of MAGEA1, CTAG1, CTAG2, SSX1, and SSX4 in patients with improved survival. We suggest that these patients' prolonged survival is attributed to the melanoma immunogenicity potentiated by the CTAs and augmented by the vaccination procedure.

Lastly, our data implies that patients who had been immunized against the autologous tumor prior to receiving ipilimumab survived longer than patients who did not receive an autologous melanoma vaccine. The precise mechanism of action of ipilimumab is not completely clear. CTLA-4 receptor blockade prevents inhibition of activated effector lymphocytes and selective increase in the ratio between T effectors and regulatory T cells within the tumor [52]. In patients with preexisting immune response, the removal of inhibitory signals boosts antitumor activity [51], leading to the hypothesis that prior vaccination might induce in a subset of patients a population of specific antitumor cytotoxic T cells and a potent immune response following immune checkpoint blockade. In this respect, it was encouraging to note that in a cohort of vaccinated patients that had received ipilimumab, the 3-year survival was 46%, compared with 19% for the nonvaccinated patients (*p* = 0.007).

## 5. Conclusions

In this noncontrolled phase II study we have shown that adjuvant treatment with autologous melanoma vaccine yields overall and disease-free survival rates that are not inferior to those obtained with interferon alpha. In a subgroup of patients who attained a strong positive skin response to unmodified autologous tumor, survival rates were exceptionally good, with a 5-year OS of 75%. Increased expression of CTAs by the tumor correlated with improved survival. Lastly, improved survival time following ipilimumab treatment was observed for patients who had previously been vaccinated. Thus, we suggest that autologous melanoma vaccine induces protective immunity and may offer leverage for other immunotherapies.

## Figures and Tables

**Table 1:** Patients characteristics.

Patients	126

Age, median (range)	59 (15–86)
Gender	
Male	69 (55%)
Female	57 (45%)
Primary melanoma	
Breslow	Median 3.05 mm (0.2–20)
Cutaneous	76 at least
Acral	10
Mucosal	0
Unknown	11
Ulcerated	29/58 documented
AJCC stage	
IIIB	57 (45%)
IIIC	69 (55%)
Satellites	22 (17%)
Number of involved LNs, mean (range)	2.4 (0–17)
Adjuvant radiotherapy	56 (44%)

**Table 2:** Clinical data and ipilimumab treatment results of 70 patients with AJCC stage IV melanoma who received or did not receive melanoma vaccine.

Prior vaccination	Yes		No		*p* ^*∗*^
	*n*	%	*n*	%	
Patient number	35	50	35	50	
M stage					
M1A	8	23	3	9	*0.3*
M1B	8	23	10	28	
M1C	19	54	22	63	
Dose					
3 mg/kg	30	86	32	91	*0.45*
10 mg/kg	5	14	3	9	
Reinduction	4	12	3	9	
Treatment stopped for toxicity	5	14	2	6	
Objective response					
CR	3	9	3	9	*0.03* ^*∗∗*^
PR	10	29	4	11	
S	6	17	1	3	
P	16	46	27	77	
Survival data					
Median OS (months)	31 (3–47)		9 (2–50)		*0.007*
3-year survival rate	46%		19%		

CR: complete response, PR: partial response, S: stable disease, and P: progression.

^*∗*^Pearson chi square (two-sided) test.

^*∗∗*^ *p* value for progression (P) versus any benefit (CR, PR, and S).

**Table 3:** Patients survival data.

	Number (%)	1 year (%)	2 years (%)	5 years (%)	Median (mo, 95% CI)	*p* value
*Overall survival*						
All^*∗*^	126	93	77	54	88 (40–137)	
DTH ≥ 15 mm	48 (40)	100	93	75	Not reached	
DTH < 15 mm	71 (60)	89	70	44	45 (13–20)	**<0001**
DTH ≥ 10 mm	75 (63)	97	83	64	181 (75–287)	
DTH < 10 mm	44 (37)	87	66	32	41 (23–59)	**0.003**
*Disease-free survival*						
All^*∗∗*^	107	58	45	34	18 (5–31)	
DTH ≥ 15 mm	36 (36)	74	53	47	36 (0.00–87)	
DTH < 15 mm	65 (64)	55	42	26	15 (7–23)	**0.27**
*Adjuvant radiotherapy* Overall survival						
All	56	90	69	41	43 (27–59)	**0.055**
DTH ≥ 15 mm	22 (42)	100	87	58	111	
DTH < 15 mm	31 (58)	84	56	33	31 (20–42)	**0.024**

^*∗*^DTH data was available for 119 of 126 patients.

^*∗∗*^DTH data was available for 101 of 107 patients with recorded disease-free survival.

**Table 4:** Selected genes expressed on melanoma cells which correlate with overall survival. The genes were depicted by Maxstat-package utilizing a Cox-regression analysis followed by rank statistics to determine the best cut-off value which separates patients into favorable versus unfavorable survival groups. ↑ = higher expression correlates with prolonged survival time; ↓ = higher expression correlates with decreased survival time.

Gene	↑/↓	Full name	Maxstat cut point	Maxstat *p* value	CoxPH hazard ratio	CoxPH *p* value
MICA	↑	MHC class I-related chain A (NKG2D ligand)	7.8	0.018	0.5	0.015
CTAG2	↑	Cancer testis antigen 2 (LAGE-1, NY-ESO-2)	5.9	0.014	0.58	0.015
SSX4 /// SSX4B	↑	Synovial sarcoma, X breakpoint 4	3.9	0.008	0.58	0.017
TGFA	↑	Transforming growth factor *α*	4.08	0.011	0.66	0.018
KIR3DX1	↑	Killer cell Ig-like receptor	4	0.023	0.56	0.02
MAGEA1	↑	Melanoma antigen family A, 1	5.52	0.042	0.69	0.028
SSX1	↑	Synovial sarcoma, X breakpoint 1	3.28	0.035	0.63	0.043
SMAD1	↓	SMAD family member 1	5.5	0.046	2.58	0.0002
HSPH1	↓	Heat shock 105 kDa/110 kDa protein 1	10.7	0.008	1.9	0.007
